# Public Attitudes toward Animal Research: A Review

**DOI:** 10.3390/ani4030391

**Published:** 2014-06-30

**Authors:** Elisabeth H. Ormandy, Catherine A. Schuppli

**Affiliations:** Animal Welfare Program, University of British Columbia, 2357 Main Mall, Vancouver, British Columbia, V6T 1Z4, Canada; E-Mail: schuppli@mail.ubc.ca

**Keywords:** animals and society, animal experimentation, governance, public engagement

## Abstract

**Simple Summary:**

Public engagement on issues related to animal research, including exploration of public attitudes, provides a means of achieving socially acceptable scientific practice and oversight through an understanding of societal values and concerns. Numerous studies have been conducted to explore public attitudes toward animal use, and more specifically the use of animals in research. This paper reviews relevant literature using three categories of influential factors: personal and cultural characteristics, animal characteristics, and research characteristics.

**Abstract:**

The exploration of public attitudes toward animal research is important given recent developments in animal research (e.g., increasing creation and use of genetically modified animals, and plans for progress in areas such as personalized medicine), and the shifting relationship between science and society (*i.e.*, a move toward the democratization of science). As such, public engagement on issues related to animal research, including exploration of public attitudes, provides a means of achieving socially acceptable scientific practice and oversight through an understanding of societal values and concerns. Numerous studies have been conducted to explore public attitudes toward animal use, and more specifically the use of animals in research. This paper reviews relevant literature using three categories of influential factors: personal and cultural characteristics, animal characteristics, and research characteristics. A critique is given of survey style methods used to collect data on public attitudes, and recommendations are given on how best to address current gaps in public attitudes literature.

## 1. Introduction

The use of animals in research fosters a diverse range of attitudes, with some people expressing desire for complete abolition of animal research practices, while others express strong support [1,2,3,4]. However, as Knight *et al.* [5] point out, the fundamental arguments used to oppose or support animal research have shifted little over time: typically, those who oppose animal research tend to focus on animal welfare and the suffering of the animals involved, whereas those who are involved in research (e.g., scientists, researchers) tend to base their arguments on the benefits of their work and the lack of alternatives to animal models [6,7].

In previous public attitudes literature, there is often no distinction made between different types of animal use and there appears to be an underlying assumption that people’s attitudes are uni-dimensional [8]. Typically, public attitudes studies involve the use of survey style methods; however, some studies do not disclose all the methodological details of the survey [9], and in some cases the questions that make up these surveys are worded in biased ways, thus compromising the value of the results.

The case is often made that the public does not have enough background knowledge to be involved in discussions or engagement exercises about animal research—the so-called deficit or ‘Enlightenment’ model [10]. Whilst having some support in studies that show a relationship between familiarity with science and support for animal research, e.g., [11,12,13,14,15], the deficit model has nevertheless been widely criticized. Indeed, one study has shown that as knowledge increases members of the public may become less supportive, particularly if the topic under discussion is considered morally contentious, e.g., [16]. Other studies have echoed this and found that in some cases familiarity with animal research was associated with lower levels of support, e.g., [12,14,17,18]. Furthermore, some authors propose that science and society cannot feasibly be separated, and have called for the democratization of scientific practice [10,19,20]. Since there are shifts toward the democratization of science [21], it becomes increasingly important to understand public attitudes toward scientific practices that invoke polarized opinion or might be considered morally contentious, such as animal research, and to develop novel mechanisms for public engagement on such issues.

The term ‘attitude’ has been used to refer to “the evaluation of an object, concept, or behaviour along a dimension of favour or disfavour, good or bad, like or dislike” [22] (p. 3). Attitudes are distinct from, but related to, people’s beliefs and values. It is postulated in the expectancy-value model [23,24] that attitudes are formed through a person’s accessible beliefs about an object, where a belief is defined as “the subjective probability that the object has a certain attribute” [22]. Azjen and Fishbein [22] (p. 4) give an illustrative example: “a person may believe that exercise (the attitude object) reduces the risk of heart disease (the attribute).” An important implication of the expectancy-value model is that attitudes towards an object are formed automatically and inevitably as we acquire new (and pertinent) information about an object’s attributes, and as the subjective values of these attributes become linked to the object [24]. Therefore, assessing people’s attitudes towards animals and animal research can tell us more about whether different types of animal research are normatively considered ‘good’ or ‘bad’ at both a personal and societal level.

There are several factors that previous literature has shown to influence people’s attitudes towards animals, and animal-based research specifically (as identified by Knight and Barnett [8]): personal and cultural characteristics, animal characteristics, and research characteristics. By exploring these influential factors in detail the following review provides an update on the survey-based public attitudes literature that was reviewed a decade ago by Hagelin *et al.* [4]. The authors then go on to discuss shortcomings associated with survey style methods in more depth. Finally, in light of this critique, the paper makes recommendations on how gaps in this growing literature can be addressed to move toward more sound models of public engagement.

## 2. Personal and Cultural Characteristics

In order to understand different attitudes toward the human use of animals, and their use in research specifically, many studies have focused on personal characteristics: that is, things about a person that may influence their decision on whether to support or oppose the use of animals in research. The personal characteristics discussed below include: age, sex, rural *versus* urban background, experience of animals/pet ownership, and religion. Also discussed are factors based more on a person’s beliefs and potentially shaped by personal characteristics: vegetarianism, and belief in animal mind.

### 2.1. Age

It has generally been reported that moral acceptance of the use of animals in research is positively correlated with age [4]. In their 1981 study [25], Kellert and Berry suggest that younger people are more opposed to animal use than older people. The authors go on to describe how older males presented a more instrumental view toward animals, suggesting that older people tend to emphasize the practical value of animals. Other studies have echoed this finding [17,26,27,28]. However, some studies have found, conversely, that younger participants are more supportive of animal-based research that older participants, e.g., [11]. The effect of age on attitudes toward animals may be a cohort effect, where people with a shared history are more likely to share beliefs and attitudes [29], or may be also be related to attitudinal change with age [30].

### 2.2. Sex

Sex identity has been consistently found to relate to attitudes toward the treatment of research animals (and animals in general), with virtually all studies reporting that women are more likely to object to animal use [12,25,26,31,32]. A lower proportion of women accept the use of animals in research compared to men [27,33,34,35,36,37] and most studies of the animal protection movement have found that women activists outnumber men by a ratio of two or three to one [38,39,40]. The effects of sex identity on attitudes toward the use of animals in research are consistent across many studies, with differences between males and females extending to at least 15 different countries [14]. Pifer [15] reported that, among a range of predictors, sex identity was the strongest correlate of opposition to animal research.

It might be that females are less supportive of animal use because they are more likely to attribute mental states to animals, and more likely to have a sympathetic reaction if they believe that animal use will cause some kind of pain or distress to animals [18]. Indeed, males have been shown to present lower levels of belief in the mental abilities of animals compared to females [41] (see later paragraph for a discussion of belief in animal mind). In addition Kellert [42] reported that men exhibited more “dominionistic” attitudes toward the environment, while women exhibited more “moralistic” attitudes, a difference that might also explain sex difference in attitudes toward animal use.

Rather than characterizing people strictly by biologically determined sex, others have examined sex role orientation (SRO) in relation to attitudes toward the use of animals in research [43,44]. Herzog *et al.* [43] suggest that differences in attitudes are associated with feminine *versus* masculine SRO, with people who identify as more feminine being generally less supportive. However, Peek *et al.* [44] speculate that sex differences differ not as a result of SRO, but because of the structural location of females in society (*i.e.*, females may perceive themselves and animals to have similar positions in society; [45]). Similarly, women’s social positions may also lead to greater concern for animals. For example, Kendall *et al*. [29] argue that women are typically primary family caretakers (and so are more likely to take on nurturing roles), and may be more likely to engage in household tasks that put them in more direct contact with animals.

### 2.3. Rural versus Urban Background

Some studies have shown that people with a rural background have a greater acceptance of animal use than urban people, and greater support for animal experimentation [14,46,47]. This finding suggests that rural and urban places provide distinct opportunities for contact and relationships with animals, as well as diverse cultural experiences that shape and strengthen people’s attitudes about animals [29]. Animal use often differs in urban and rural regions [39]. The instrumental relationships with animals that are associated with rural settings might shape an individual’s attitudes toward animals in different contexts, including animal research. A cross-cultural study of people’s attitudes toward the use of animals in research [14] found that there was a link between a nation’s level of industrialization and urbanization and attitudes toward animal research. For instance, the two least industrialized countries within the European Community had the highest level of support for animal research. Crettaz von Roten [13] also found differences in acceptance of animal research between European countries, with industrialized countries (*i.e.*, countries where labor is more physical in nature) displaying higher levels of approval of animal research than post-industrial countries (*i.e.*, countries where labor is more mental in nature). Pifer *et al.* [14] suggest that countries that have closer relationship with the land have more pragmatic and utilitarian attitudes about animals, such that the use of animals by humans in not seen as contentious. In developed countries urban people may never come into contact with the animals they eat; instead, animals are more likely to be companions and part of the family [39]. Perhaps for this reason, urban residence has been found to be related to greater concern for animal well-being [31,46,48].

### 2.4. Experience with Animals

Attitudes toward the human use of animals can also be shaped by a person’s previous or existing experience of animals [8,35]; for example, Driscoll [26] found that pet owners rated animal-based research as less acceptable than did non-pet owners. This finding is also echoed in other studies that showed that pet owners form an attachment with their animals, and that this strengthens a general positive attitude toward other animals [49,50,51,52]. According to ‘contact theory’, e.g., [53], contact with members of an ‘outgroup’ (e.g., non-human animals) can lead to a mutual understanding and decreased prejudice toward that group. Contact may also foster emotional attachment and empathy toward animals [54,55,56,57]. This may explain why positive experiences of animals promote affection and positive attitudes toward animals in general, which is in conflict with utility or instrumental uses of animals, such as research animals [58]. Thus pet ownership, or other positive experiences of animals may increase people’s opposition to animal research. Conversely, a negative encounter with an animal may equally shape people’s views, making them more supportive of animal use [59]. In addition, the type of contact that an individual has with animals may also influence their attitudes towards animals: as previously mentioned, contact with animals through circumstances such as farming may promote a more instrumental view towards animals, rather than one based on companionship.

### 2.5. Religion

Religion can influence how people view and relate to animals. For example, Christianity has been shown to be positively associated with support for the use of animals in research [60]. Driscoll [26] found differing views across different Christian denominations: persons reporting no religious affiliation or an affiliation with the Catholic church rated various examples of animal-based research as less acceptable than did persons reporting a traditional Protestant affiliation. There are, of course, also specific animal species that are either revered (e.g., cows in Hinduism) or avoided (e.g., pigs in Judaism) in different religious traditions. This may in turn affect people’s willingness to support or oppose the use of certain species for research purposes.

### 2.6. Personality

An individual’s personality type, and the way in which people morally evaluate situations can influence their willingness to support animal research. Previous literature has classified people into four ethical perspectives: absolutists (high idealism, low relativism), situationists (high idealism, high relativism), exceptionists (low idealism, low relativism), and subjectivists (low idealism, high relativism) [61]. Working with this framework, Galvin and Herzog [62] have illustrated that absolutism (high idealism) is high amongst animal activists, as opposed to subjectivism (low idealism), which was low. In a separate study, Galvin and Herzog [63] also showed that idealism was high amongst participants who rejected hypothetical animal research proposals. These findings were further echoed in a study by Wuensch and Poteat [64] in which different types of animal research proposals were approved by participants who were significantly less idealistic and significantly more relativistic. Overall, evidence to date suggests that support for animal research is negatively associated with personality types that tend towards idealism, and positively associated with relativism.

### 2.7. Vegetarianism and Animal or Environmental Advocacy

Vegetarianism has been associated with lower acceptance of the use of animals in research compared to non-vegetarianism [11,17,50]. Demand for particular types of food is influenced primarily by social and psychological factors such as beliefs, attitudes, norms, and values [46], and vegetarianism is related to value orientations such as an increase in altruistic values and a decrease in traditional (*i.e*., instrumental) values [65]. Moreover, vegetarianism is likely to relate to a wider ideological perspective in terms of the ‘world view’ or ‘ethical ideology’ held by people [27,66,67]. So, rather than being a predictor of attitudes toward animals *per se*, vegetarianism is an action or behaviour that results from a particular attitude toward animals. This attitude may be generalized into a broader concern with animal rights, protection or welfare, due to underlying beliefs, meaning that vegetarian individuals are more likely to oppose the use of animals in research.

In a similar vein, an interest in environmental issues (which may also be linked to vegetarianism) is negatively related to support for animal research [12]. Studies have shown that people who are politically left-wing-oriented are less supportive of animal experimentation. This finding may also be explained by differences in people’s worldviews or ethical ideologies [9,66,68], because attitudes toward animals are closely related to attitudes toward other political and social matters [27].

### 2.8. Belief in Animal Mind

“Belief in animal mind” (BAM) is the term used to describe people’s belief in the mental abilities of animals. Does one believe that animals are self-aware, capable of solving problems, or experiencing emotions such as fear, sadness, happiness and pleasure? [18,41]. BAM is a relatively consistent predictor of attitudes toward the human use of animals [18,41,46,69], and in one small qualitative study BAM appeared to explain more of the variation in people’s attitudes than personal characteristics, such as sex [8]. BAM negatively correlates with support for animal use and positively correlates with concern for animal welfare and humane behaviour toward animals [8,12], and empathy toward other humans and animals [46]. If one believes that certain species are likely to experience internal thoughts and feelings, then subjecting them to discomfort as part of animal-based research may seem unacceptable. This line of reasoning would suggest that people should be less accepting of research using species rated highly in BAM, particularly non-human primates. However, a study by Knight *et al.* [5] showed that more support was expressed for the use of monkeys in medical research compared to other animals, such as dogs, cats, rabbits, guinea pigs, rats and mice. In this study it was scientists (rather than lay persons or animal welfarists) who indicated strong support for the use of monkeys in research. Knight *et al.* [5] show that, despite attributing ‘animal mind’ to monkeys, scientists’ perception was that monkeys are more appropriate animal models for medical research practice. This finding shows that, in some cases, BAM may be trumped by other factors (such as perceived benefit or necessity of research).

## 3. Animal Characteristics

While most studies have focused on personal and cultural characteristics to explain variation in attitudes, factors relating to animal characteristics also influence people’s view on this subject. The animal characteristics discussed below include species, sentience, neoteny/appeal and genetic modification.

### 3.1. Species, Sentience and Appeal

People hold different attitudes toward animal use depending on the species involved [26,41,70]. People tend to rate animals classed as pets (e.g., dogs and cats) or non-human primates as having higher mental abilities compared to other species such as fish or mice [41,71]. People are more supportive of using smaller-brained animals such as mice and rats [71], and less supportive of using animals classed as pets [26], and animals believed to have ‘higher’ mental abilities enabling them to use tools, solve problems, and be self aware [8,41]. Therefore, the same person may support the use of mice and rats for dissection purposes, but not support the use of chimpanzees, cats or dogs for the same purpose. In a recent study involving interviews with members of animal care committees (responsible for the ethical review of research proposals involving the use of live animals) Schuppli [69] reported that committee members were less comfortable with research using non-human primates and companion animals. Different views regarding species may be due to a belief in the mentality of different species, or their human-like qualities in terms of human experience [69,72] as well as other factors such as personal affection for particular kinds of animals, or individual animals [73], the special consideration given to certain species based on the relationship we typically have with those animals [35,74], where the species falls on the phylogenetic scale [75], or their ‘cuteness’ or attractiveness [4,8,41]. From literature on public attitudes toward species conservation, it has also been shown that animals that retain a neonatal appearance (neoteny) are more likely to be supported in conservation efforts [76,77].

However, it is not always the case that animal research using species that are lower on the phylogentic scale is more acceptable. In a study asking participants about their willingness to support the use of animals to create models of skin cancer, there was no species effect of switching from zebrafish to mice (despite predictions that support would drop when fish were replaced with mammals [78]). Attitudes toward the use of different species in research may also change as we learn more about animal behaviour and welfare; for example, recent research suggests that fish (that are often considered an acceptable replacement for mammals in research [79,80,81]) have the capacity to feel pain [82,83].

### 3.2. Genetic Modification

Public views toward the genetic modification of animals tend to be complex, but predominantly negative [84]. Genetic modification of animals presents new challenges in terms of maintaining public acceptance of animal-based research. Some members of the public express grave concern for the ‘unnaturalness’ of genetic modification and its potential to lead to unknown consequences [17,68,85]. Indeed, people’s perception of what is “natural” has been shown to decrease with the alteration of genetic material through genetic engineering [86]. In his 2001 study, Macnaghten [85] found considerable concern about genetic modification and the uses to which genetically modified (GM) animals might be put. Participants in his focus-group study showed a “reaction against the proposed technology as intrinsically a violation of nature and transgressive of so-called natural parameters” [85] (p. 25)—what might be called the “yuk response” [87]. Such findings are echoed in other studies, e.g., [26,88]. Another primary concern that has emerged is that genetic modification might lead to unexpected (and potentially bad) consequences; indeed, one aspect of the unease about GM animals is a fear that nature might ‘bite back’ [84,89]. In addition to these main arguments in opposition to GM, a more recent study by Macnaghten [89] shows an emerging concern from the public about the increase in the numbers of animals used in research due to the currently inefficient and unpredictable nature of the genetic modification process. This sentiment also emerges in studies by Schuppli *et al.* [74] and Ormandy *et al.* [90] who argue that the creation and use of GM animals challenges the Three Rs principles (replacement, reduction, refinement), particularly reduction.

## 4. Research Characteristics

The characteristics of the research that an animal will be involved in can also influence people’s decisions about whether to support or oppose the research. The research characteristics discussed below are: the purpose of the research, the level of invasiveness (or harm) that the animal will experience, and availability of non-animal alternatives.

### 4.1. Type of Research

It is common that medical experiments involving animals are more positively regarded than experiments for cosmetics testing. For example, Aldhous *et al.* [91] found that whether or not mice were subjected to pain, illness, or surgeries, people were more likely to disapprove if the experiment was designed to test the safety of a cosmetics ingredient than if it tested the safety and effectiveness of a drug or vaccine, and this result was echoed in numerous other studies [8,14,26,64,69]. Conversely, Schuppli and Weary [11] found that participants in an online public engagement study were more supportive of the use of pigs in environmental research (to reduce agricultural pollution) than for biomedical research (to decrease rejection rates in organ transplantation). However, the purpose of the research may be trumped by other influential factors. For example, non-animal alternatives to the biomedical research scenario used in the study by Schuppli and Weary [11] (e.g., increasing human organ donations) may be seen as a more viable option. It would appear that people’s attitudes toward experiments involving animals are likely to change depending on the beneficiary, purpose or necessity of the research. As noted by Henry and Pulcino [92] “the literature suggests that animal research that is viewed as providing tangible, meaningful benefits to humans is considered more acceptable than animal research that is viewed and less beneficial or necessary.”

### 4.2. Availability of Alternatives

The perceived necessity of animal research ties into the availability of non-animal alternatives, with research that is deemed unnecessary being less favoured. For example, Stanistreet and Spofforth [93] found that participants were less supportive of the use of animals in research that was viewed as “non-necessary” than research that was viewed as “necessary.” It seems that the availability of non-animal alternatives, or a belief that alternatives exist, may be particularly influential on people’s attitudes toward the use of animals in research, e.g., [4]. Two studies in particular illustrate that when non-animal alternatives are available, there is higher level of opposition. Research by Knight *et al.* [18] showed that animal use was most likely to be supported when participants perceived there to be no other choice than using animals. However, Knight *et al.* [18] also found that their participants (nine men, eight women) could seldom think of alternatives to using animals in research and in teaching, and so they believed that there was little choice other than using animals. In a follow up study, Knight *et al.* [5] showed that different attitudes toward animal experimentation between scientists and animal welfarists could, in part, be explained by differing beliefs in the availability of non-animal alternatives.

### 4.3. Level of Harm

Invasiveness, or level of harm that the animals experience during a given experiment has also been shown to influence people’s support of animal-based research [33,37]. Richmond *et al.* [94] found that the most common objection to animal experimentation is related to whether animals experience pain and suffering. In fact, a review by Hagelin *et al.* [4] illustrated that survey respondents are less likely to support animal research if the words “pain” or “death” are used. In a more recent study [92] results indicated that participants were more opposed to biomedical research that resulted in harm to animals.

In addition, Bateson [95] has made the argument that animal suffering (level of harm) should be weighed against the importance of the research, and the likelihood of benefit when making decisions about whether animal research should proceed. As described in the subsections above, these factors (especially the importance of the research) are also important to members of the public.

## 5. Other Variables

There are other variables that may affect people’s attitudes towards animals, or animal research, that do not fit neatly into the three categories above. In particular, the effect of social media, and the living conditions of animals in laboratories have been shown to have an effect on people’s attitudes.

### 5.1. Effect of Social Media

The use of social media by animal rights organizations has been successful in raising public awareness of certain issues related to animal research [96]—this is further illustrated by the large memberships of social media groups with an animal rights or welfare focus (e.g., PETA currently have over 2 million Facebook group members). To the author’s knowledge no academic literature to date has explicitly tested the effects of social media on people’s willingness to support animal research. However, one study by Kruse *et al.* [97] documented how pro-research efforts get more positive attention in social media. The authors of this study go on to argue that members of the public are the most easily influenced by social media because they are not well-informed about animal research. A different study [98] documented how public attitudes towards California’s cougars were shifted and reflected over a decade (1985–1995) through print media.

### 5.2. Living Conditions of Laboratory Animals

In several different online engagement studies, participants were more willing to support animal research provided that their concerns about animal welfare (including the day-to-day care and handling of animals) were addressed [17,78,88]. These findings indicate that the living conditions of animals kept for research purposes can affect people’s attitudes towards animal research, and if animals are well-housed and cared for, people’s support for animal research will perhaps increase.

## 6. Critique of Existing Methods of Public Attitudes Assessment

There is a growing body of literature related to public attitudes toward animal use in general, and animal research more specifically. However there are potential shortcomings that should be addressed for future studies. Three primary shortcomings are discussed below: use of college students as participant samples, use of general questions about ‘animal use’ rather than specific questions about different types of animal use (or even different types of animal research), and use of Likert scales or rating scales that do not allow for more qualitative reasoning.

While numerous previous studies have engaged with a broader public membership when assessing attitudes towards animals and animal research, e.g., [13,26,35], many others have used undergraduate students (usually majoring in psychology) for their sample populations, e.g., [31,62,64,99]. In fact, Herzog and Dorr [100] examined 15 issues of Society and Animals published between 1993 and 1998 and found that, “the data in 11 of these articles were obtained using undergraduates. Of these, one article did not specify the source of the students, one used education students as subjects and the other nine were based in students taking psychology classes” [100] (p. 2). Notably, using a large national sample, Kellert [101] and Kellert and Berry [25] reported that both education and age were related to knowledge and attitudes toward animals. This suggests that college students, being both young and educated, are likely to be more concerned about animals than the general public. Given that the regulation of animals in research was developed, in part, in response to public concerns, it is pertinent that new ways of assessing attitudes toward the use of animals in research are developed that reflect a diversity of views, rather than limiting the breadth of studies by relying on convenience sampling of students. As further pointed out by Herzog and Dorr, “undergraduate psychology majors are a narrow source of information on human/animal relationships” [100] (p. 2). This is echoed in a recent article in the Economist [102], which highlights the challenges to using undergraduate students as a source of information and explores the benefits of crowd sourcing (e.g., the use of Amazon’s Mechanical Turk platform to recruit survey participants). The primary benefit to crowd sourcing is the diversity of participants: there is less reliance on information provided by participants from western, educated, industrialized, rich and democratic subsets of the world population.

A second shortcoming is that most studies have asked rather general questions about animal use. Kellert and Berry [25], Driscoll [26] and Knight *et al.* [18] have identified this problem, showing that people have strong likes and dislikes for different kinds of animals, and multidimensional views regarding different types of animal use. To ask someone to agree or disagree with a statement such as “it is alright to do research on animals” is ambiguous. It may be that only people with more extreme views will disagree with this statement because it does not specify what kind of research, or perhaps more importantly, what kind of animals are involved. 

Research animal use is changing, particularly as a result of increasing use of technologies such as genetic modification [90] and ethyl-N-nitrosourea (ENU) mutagenesis (a commonly used method of chemically inducing mutations, particularly in mice [103] and zebrafish [104]) to create animal models of disease. So far, research exploring public attitudes to genetic modification of animals has mainly focused on farm animals, rather than laboratory animals that are used in much greater numbers. Only one study to date has explored people’s views toward ENU mutagenesis [78]. In addition, new developments in areas of personalized medicine, particularly oncology, may pose new challenges. For example, a patient with a tumour might be able to have tumour samples taken and implanted into animal hosts (e.g., mice) so that a range of treatments can be tested, and a better targeted therapeutic treatment for the patient developed [105]. Such procedures will likely increase animal numbers and may also require alterations to the current process of animal protocol review and approval, as well as perhaps introducing a more personal, direct involvement in the public’s role in animal use.

A third shortcoming is that many of the studies cited above were performed using methods that asked participants to respond on a scale (e.g., Likert scale, rating or preference scale), or asked questions requiring a simple “Yes” or “No” response, without any insight into the reasoning that may have led to these responses. Participants are constrained in their choice of answers by the options provided by the researcher (which may lead to researcher bias) [106] and are unable to provide any qualification to explain their response. The exploration of people’s reasons for their “Yes”/”No” or Likert scale responses is important. The shortcomings of restricted response options can be addressed if questions are designed with sufficient understanding of the topic: being able to ask meaningful questions that allow people to demonstrate their reasoning. Often such in-depth understanding is developed from initial qualitative research, where the quantitative research is used to confirm the findings. When restricted response options do not allow for consideration of what people’s concerns are (e.g., why they might be opposed to certain types of research), it is difficult for policy makers to understand the nuance in attitudes in order to make progress in addressing societal concerns.

Aside from academic research, regular national opinion polls often ask questions about people’s level of support for animal research. These polls can be valuable in tracking attitudes over time, and they invite broader perspectives from a wider and more representative sample population; however the polls remain subject to the prior criticism of using fixed response options for participants to choose from. As further pointed out by Hobson-West [107] care should be taken when referring to others’ interpretation of national opinion polls, since the same posed can be used as evidence by both sides of the polarized debate about animal research.

## 7. Addressing the Gaps through Better Public Engagement

Pytlik Zillig and Tomkins [108] argue that public engagement is a valuable means to provide societal perspectives concerning the political, legal, ethical, and other impacts of scientific and technological research. Changes in societal attitudes often result in a push to improve animal-related regulation and public policy [109]. However, current mechanisms for including public opinion in animal research policy may be lacking. One recent article highlights the secrecy surrounding animal research [110] while another [111] draws attention to some of the problems that might be encountered if decisions about animal research are not opened up to a wider community. The case study by Lyons [111] warns against the formation of policy communities with exclusive membership that “tend(s) to produce outcomes that consistently favour network members at the expense of excluded groups” [111] (p.357). In the article, Lyons describes a specific area of research (xenotransplantation between pigs and primates) in which, to the detriment of the animals involved, decisions were made without input from experts and stakeholders outside the policy community, and without wider public engagement. Such activities go against the increasing democratization of science and science policy [10,19,21], and highlight the need for wider public engagement, especially for research that is considered to be contentious. Therefore, it is important for governing bodies to assess public opinion about animal-based research, and to engage a variety of different stakeholders, including the public, when developing animal policy.

One approach to improving public engagement on animal research issues is to conduct further empirical studies that explore public attitudes toward animal research in ways that correct for some of the criticisms outlined in this paper. For example, studies that: (1) avoid reliance on convenience sampling of students, and ensure that participants reflect a diversity of views; (2) use a well-planned experimental framework that allows exploration of not only where people draw the line in terms of what they are willing to accept, but also why; and (3) focus on gaining a better understanding of public attitudes toward specific (rather than general) aspects of animal research; for example, attitudes toward emerging technologies (like genetic modification or other genetic alteration techniques) and the most commonly used species in research (zebrafish and mice), as well as the regulatory systems that oversee animal research.

## 8. Summary

Various factors influence people’s views toward the use of animals in research, and these can be categorized into: (1) personal and cultural characteristics; (2) animal characteristics; and (3) research characteristics. Understanding public attitudes toward the use of animals in research will facilitate the growing trend toward more openness and democratization of scientific research, and ensure that scientific practice (including animal research) remains in step with societal values. In turn, evaluating societal values and addressing societal concerns is important, as the public is often claimed to be the key beneficiary of the resulting therapeutic products that are developed and tested.
